# *Tessaria absinthioides* (Hook. & Arn.) DC. (Asteraceae) Decoction vs. Metformin: Effects on Glucose Homeostasis and Behavior Under Chronic Sucrose Exposure

**DOI:** 10.3390/plants15182872

**Published:** 2026-09-19

**Authors:** Héctor Coirini, María Sol Kruse, Mario J. Simirgiotis, Jessica Gómez, Alejandro Tapia, Mariana Rey

**Affiliations:** 1 Laboratorio de Neurobiología, Instituto de Biología y Medicina Experimental (IBYME-CONICET), Ciudad Autónoma de Buenos Aires C1428ADN, Argentina; hcoirini@ibyme.conicet.gov.ar (H.C.); sol.kruse@conicet.gov.ar (M.S.K.); 2 Instituto de Farmacia, Facultad de Ciencias, Campus Isla Teja, Universidad Austral de Chile, Valdivia 5090000, Chile; mario.simirgiotis@uach.cl; 3 Center for Interdisciplinary Studies on the Nervous System (CISNe), Universidad Austral de Chile, Valdivia 5090000, Chile; 4 Instituto de Biotecnología-Instituto de Ciencias Básicas-Departamento de Ingeniería Agronómica, Universidad Nacional de San Juan (UNSJ), San Juan J5400ARL, Argentina; jesicagomez674@gmail.com (J.G.); atapia@unsj.edu.ar (A.T.); 5 Consejo Nacional de Investigaciones Científicas y Técnicas (CONICET), Ciudad Autónoma de Buenos Aires C1425FQB, Argentina

**Keywords:** *Tessaria absinthioides*, exploratory behavior, risk-assessment behavior, glucose homeostasis, Asteraceae, caffeoylquinic acids, UHPLC-PDA-OT-MS/MS

## Abstract

*Tessaria absinthioides* (Hook. & Arn.) DC. (Asteraceae) decoction (DTa) is used to treat hypercholesterolemia, diabetes and digestive disorders in the traditional medicine of Argentina, Bolivia, Chile, and Uruguay. The present study evaluated the effects of DTa on glucose metabolism and behavior and compared them with metformin under chronic sucrose exposure and normal conditions. Male Sprague-Dawley rats received water, sucrose, DTa (5 or 10% *w*/*v*) with or without sucrose, or metformin (12.5 or 25 mg/kg) under water- or sucrose-fed conditions for 40 days, according to the experimental design. Glucose homeostasis was assessed by fasting glycemia and a glucose tolerance test (GTT), while behavior was evaluated using the open field, elevated plus maze (EPM), and novel object location tests. Under sucrose-fed conditions, DTa was associated with lower fasting glycemia, while both DTa and metformin improved GTT, as evidenced by reduced iAUC values. Behavioral analyses revealed modulation of exploratory and risk-assessment behavior by DTa, particularly in the EPM, where DTa10 increased total distance traveled and head-dip behavior under sucrose-fed conditions. Under normal conditions, DTa10 was associated with lower fasting glucose levels without significant changes in GTT, whereas behavioral effects were mainly restricted to specific ethological parameters. No effects were detected on spatial recognition memory under either condition. In DTa, fifty-one compounds were detected by ultrahigh-performance liquid chromatography coupled to photodiode array and Orbitrap mass spectrometry (UHPLC-PDA-OT-MS/MS), of which forty-one were identified, including several caffeoylquinic acids, fatty acids, and characteristic eudesmane sesquiterpenoids. Several of these compounds have been previously associated with metabolic and neurobehavioral effects, providing further support for the pharmacological potential of DTa. Taken together, these findings highlight *T. absinthioides* as a promising source of bioactive phytocompounds with the potential to modulate glucose homeostasis and behavioral responses.

## 1. Introduction

*Tessaria absinthioides* (Hook. & Arn.) DC. (Asteraceae) is a perennial medicinal shrub (1–1.5 m tall) with tomentose stems, lanceolate serrated leaves covered by a dense tomentose indumentum, and terminal capitulate inflorescences. Native to South America, it is commonly found in arid and semiarid environments, particularly along riverbanks and irrigation channels, and is traditionally consumed as an infusion or decoction in Argentina, Bolivia, Chile, and Uruguay for the treatment of hypercholesterolemia, diabetes, and digestive disorders [1,2]. The leaves of *T. absinthioides* have also been associated with hypocholesterolemic, balsamic, and expectorant properties [3]. In addition, several reports have demonstrated antimicrobial, cytotoxic, antitumoral, and antioxidant activities [4,5]. Consistently, we previously showed that a decoction of *T. absinthioides* (DTa), prepared from plants collected in San Juan Province, Argentina, reduced total cholesterol levels, increased HDL-c, and modulated the expression of liver X receptors in a high-fat diet model [6]. These effects may be related, at least in part, to the presence of bioactive phytochemicals identified in DTa. In the same study, UHPLC-PDA-Orbitrap MS analysis identified chlorogenic acid, several di- and tri-caffeoylquinic acid derivatives, vanillic acid, flavonoids, and characteristic sesquiterpenes as major constituents of DTa [6]. In particular, chlorogenic acid and other caffeoylquinic acid derivatives have been associated with beneficial effects on glucose homeostasis in experimental models [7,8,9]. Moreover, chlorogenic and vanillic acids have also been shown to modulate anxiety-like behavior and exert neuroprotective effects in experimental models [10,11]. Together, these findings support the potential relevance of DTa phytochemicals for both metabolic and neurobehavioral regulation.

Chronic sucrose consumption has been widely used to induce metabolic alterations associated with a prediabetic state. In experimental models, prolonged sucrose exposure has been associated with glucose intolerance, insulin resistance, increased fasting blood glucose, and hypertriglyceridemia [12,13,14,15]. Moreover, prolonged exposure to sucrose has been associated with neurobehavioral alterations, including anxiety-like behavior and cognitive deficits [12,13,14,15]. Metformin is one of the most widely used pharmacological agents for the treatment of type 2 diabetes due to its well-established effects on glucose homeostasis. Using a similar experimental model, we previously demonstrated that metformin treatment prevents sucrose-induced changes in exploratory and anxiety-related behaviors in the open field (OFT) and elevated plus maze (EPM) tests, although it does not restore impairments in spatial recognition memory [16]. Despite its therapeutic benefits, metformin treatment may be associated with adverse effects, particularly gastrointestinal disturbances [17], highlighting the continued interest in identifying alternative or complementary strategies based on natural products. In this context, medicinal plants rich in bioactive phytochemicals represent promising candidates for the modulation of metabolic and behavioral alterations associated with metabolic imbalance. Evaluating these compounds under both pathological and physiological conditions may also help to distinguish context-dependent effects from broader actions on basal metabolic homeostasis and behavioral regulation.

Based on its chemical composition and previously reported metabolic effects, *T. absinthioides* emerges as a promising candidate for the evaluation of its beneficial properties under conditions of metabolic challenge. Therefore, the aim of the present study was to evaluate the effects of DTa on glucose homeostasis and behavior in rats chronically exposed to sucrose and under normal conditions, and to compare its effects with those of metformin. We hypothesize that DTa may exert beneficial effects on metabolic and behavioral parameters, particularly under conditions of metabolic stress, owing to its bioactive phytochemical composition.

## 2. Results

The results are presented separately for the two experimental settings: sucrose-induced metabolic challenge (SUC-GR) and normal physiological conditions (WAT-GR). SUC-GR included 10% sucrose (SUC), 10% sucrose prepared in 5% or 10% DTa (SDTa5 and SDTa10), and 10% sucrose plus metformin at 12.5 or 25 mg/kg (SMET12.5 and SMET25). WAT-GR included water (CON), 5% or 10% DTa (DTa5 and DTa10), and water plus metformin at 12.5 or 25 mg/kg (MET12.5 and MET25).

### 2.1. Body Weight (BW) and Beverage Intake

In SUC-GR, BW increased significantly over time (F(3.163,110.7) = 1490; *p* < 0.0001), without a treatment × time interaction or main effect of treatment (both *p* > 0.9999; Figure S1A). Beverage intake also varied over time (F(1.526,18.31) = 220.4; *p* < 0.0001), with a significant treatment × time interaction (F(6.103,18.31) = 4.393; *p* = 0.0063), although the main effect of treatment was not significant (F(4,12) = 0.4779; *p* = 0.7515). Despite the significant interaction, post hoc comparisons showed no differences among groups at any individual time point (Figure S1B).

A similar pattern was observed in WAT-GR. BW increased significantly over time (F(2.617,83.75) = 3192; *p* < 0.0001), without a significant treatment × time interaction (F(10.47,83.75) = 0.3638; *p* = 0.9626) or main effect of treatment (F(4,32) = 0.01852; *p* = 0.9993; Figure S1C). Beverage intake varied significantly over time (F(1.352,16.22) = 304.7; *p* < 0.0001), with a significant treatment × time interaction (F(5.407,16.22) = 2.934; *p* = 0.0426), but no main effect of treatment (F(4,12) = 0.5206; *p* = 0.7225). Again, post hoc comparisons showed no differences among groups at any individual time point (Figure S1D).

### 2.2. Fasting Glycemia (GLU) and Glucose Tolerance Test (GTT)

**Figure 1:**
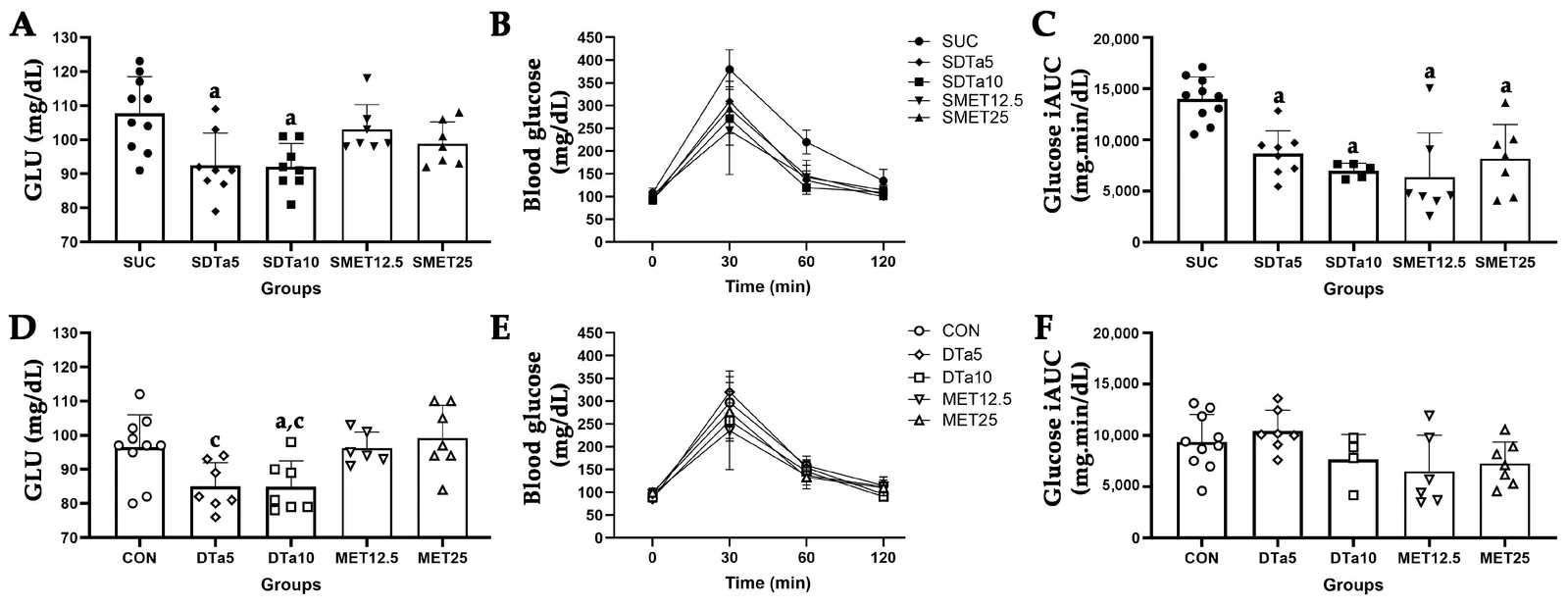
Basal glucose (GLU), glucose tolerance test (GTT) curves, incremental area under the curve (iAUC) in the SUC-GR (**A**–**C**) and in the WAT-GR (**D**–**F**). GLU and iAUC values were analyzed by one-way ANOVA followed by Tukey’s post hoc test. GTT curves were analyzed by two-way RM-ANOVA followed by Šídák’s multiple comparisons test. Sample sizes for GLU were: SUC (*n* = 10), SMET12.5 (*n* = 7), SMET25 (*n* = 7), SDTa5 (*n* = 8), SDTa10 (*n* = 8), CON (*n* = 10), MET12.5 (*n* = 6), MET25 (*n* = 7), DTa5 (*n* = 7) and DTa10 (*n* = 7). For GTT and iAUC: SUC (*n* = 10), SMET12.5 (*n* = 7), and SMET25 (*n* = 7), SDTa5 (*n* = 8), SDTa10 (*n* = 5), CON (*n* = 10), MET12.5 (*n* = 6), MET25 (*n* = 7), DTa5 (*n* = 7), and DTa10 (*n* = 4). Data are presented as mean ± SD. **^a^** *p* < 0.05 vs. the corresponding control group (SUC in SUC-GR or CON in WAT-GR); **^c^** *p* < 0.05 vs. MET25 in WAT-GR.

In SUC-GR, GLU differed among groups (one-way ANOVA: F(4,35) = 5.504; *p* = 0.0015). Tukey’s post hoc test showed lower GLU in both SDTa5 and SDTa10 compared with SUC (−14.19%; *p* = 0.0050 and −14.54%; *p* = 0.0038, respectively), whereas neither metformin dose differed from SUC (*p* > 0.05). No differences were detected between the two DTa concentrations or between the two metformin doses (all *p* > 0.05; Figure 1A). During the GTT, two-way RM ANOVA revealed a significant treatment × time interaction (F(5.565,44.52) = 4.365; *p* = 0.0019), as well as significant effects of treatment (F(4,32) = 12.73; *p* < 0.0001) and time (F(1.391,44.52) = 274.3; *p* < 0.0001). Šídák’s post hoc test showed lower glucose levels in SDTa5 than in SUC at 30, 60, and 120 min (*p* = 0.0416; *p* < 0.0001; and *p* = 0.0163, respectively), and in SDTa10 at 30 and 60 min (*p* = 0.0029 and *p* < 0.0001, respectively). SMET12.5 and SMET25 showed lower glucose levels than SUC at 60 min (*p* = 0.0075 and *p* = 0.0003, respectively). No significant differences were detected between either DTa concentration and either metformin dose at any time point (Figure 1B). The incremental area under the glucose curve (iAUC) differed significantly among groups (F(4,32) = 9.909; *p* < 0.0001). Both SDTa5 and SDTa10 showed lower iAUC values than SUC (−38.18%; *p* = 0.0033 and −49.98%; *p* = 0.0008, respectively), as did SMET12.5 and SMET25 (−54.59%; *p* < 0.0001 and −41.79%; *p* = 0.0019, respectively). No differences were detected between SDTa5 and SDTa10 (*p* = 0.8448), between SMET12.5 and SMET25 (*p* = 0.7629), or between either DTa concentration and either metformin dose (all *p* > 0.05; Figure 1C).

In WAT-GR, GLU differed among groups (one-way ANOVA: F(4,32) = 5.085; *p* = 0.0028). Tukey’s post hoc test showed lower GLU in DTa10 than in CON (−12.06%; *p* = 0.0490), whereas DTa5 did not significantly differ from CON (*p* = 0.0532). Neither metformin dose differed from CON (*p* > 0.05). Both DTa5 and DTa10 showed lower GLU than MET25 (−14.26%; *p* = 0.0215 and −14.40%; *p* = 0.0198, respectively). No significant differences were detected between either DTa concentration and MET12.5 (*p* > 0.05; Figure 1D). During the GTT, two-way RM ANOVA revealed no significant treatment × time interaction (F(5.095,36.94) = 1.917; *p* = 0.1138) or main effect of treatment (F(4,29) = 2.176; *p* = 0.0967), whereas a significant effect of time was observed (F(1.274,36.94) = 204.0; *p* < 0.0001; Figure 1E). iAUC did not show an overall treatment effect (F(4,29) = 2.642; *p* = 0.0538; Figure 1F).

### 2.3. Open Field Test (OFT), Elevated Plus Maze (EPM) and Novel Object Location (NOL)

#### 2.3.1. OFT

**Figure 2:**
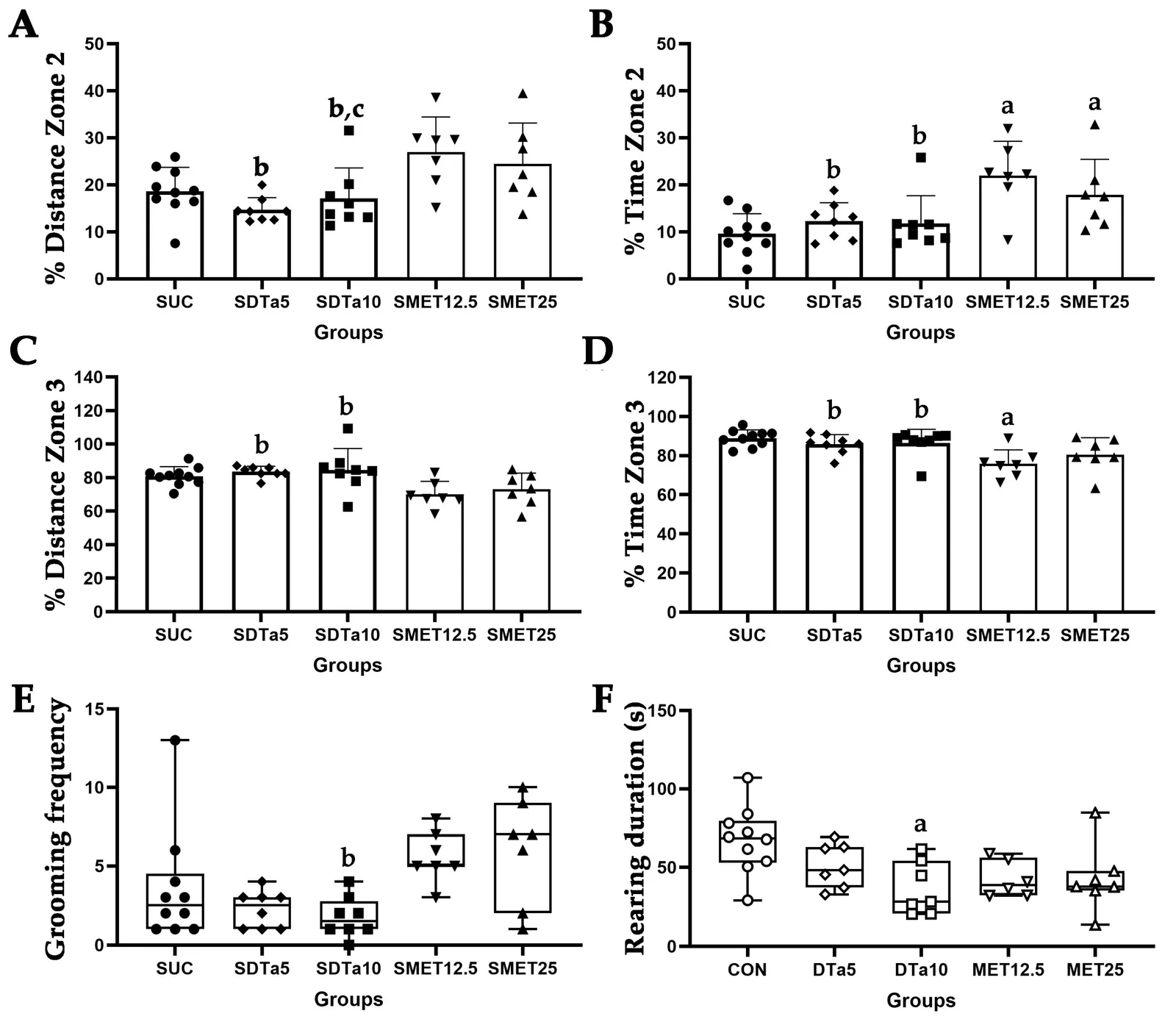
Open field (OFT) behavioral parameters in the SUC-GR: (**A**) % Distance in Zone 2, (**B**) % Time in Zone 2, (**C**) % Distance in Zone 3, (**D**) % Time in Zone 3, (**E**) Grooming frequency, and in the WAT-GR: (**F**) Rearing duration. Data in panels (**A**–**D**) were analyzed using one-way ANOVA followed by Tukey’s post hoc test, whereas Grooming frequency (**E**) and Rearing duration (**F**) were analyzed using the Kruskal–Wallis test followed by Dunn’s multiple comparisons test. Data are presented as mean ± SD for (**A**–**D**) and as median (interquartile range) for (**E**,**F**). Sample sizes were: SUC (*n* = 10), SDTa5 (*n* = 8), SDTa10 (*n* = 8), SMET12.5 (*n* = 7), SMET25 (*n* = 7), CON (*n* = 10), DTa5 (*n* = 7), DTa10 (*n* = 7), MET12.5 (*n* = 6), and MET25 (*n* = 7). **^a^** *p* < 0.05 vs. the corresponding control group (SUC in SUC-GR or CON in WAT-GR); **^b^** *p* < 0.05 vs. SMET12.5; **^c^** *p* < 0.05 vs. SMET25.

In SUC-GR, no significant differences among groups were detected in total distance traveled or in the percentage of entries into any OFT zone (all *p* > 0.05). No significant differences were observed in Zone 1 for the percentage of distance traveled or time spent (all *p* > 0.05). In Zone 2, significant treatment effects were observed for the percentage of distance traveled (F(4,35) = 5.093; *p* = 0.0024) and time spent (F(4,35) = 5.921; *p* = 0.0010). Neither DTa concentration nor metformin dose differed from SUC in the percentage of distance traveled (*p* > 0.05). However, SDTa5 and SDTa10 showed lower values than SMET12.5 (−9.23%, *p* = 0.0047 and −36.63%, *p* = 0.0322, respectively), while SDTa5 also showed a lower value than SMET25 (−40.00%; *p* = 0.0345; Figure 2A). For the percentage of time spent, SMET12.5 and SMET25 showed higher values than SUC (128.61%, *p* = 0.0011 and 86.00%, *p* = 0.0495, respectively), whereas neither SDTa5 nor SDTa10 differed from SUC (*p* > 0.05). Both DTa groups showed lower values than SMET12.5 (−44.12%, *p* = 0.0219 and −46.26%, *p* = 0.0149, respectively), with no difference between SDTa5 and SDTa10 (Figure 2B). In Zone 3, significant treatment effects were observed for the percentage of distance traveled (F(4,35) = 4.494; *p* = 0.0049) and time spent (F(4,35) = 5.347; *p* = 0.0018). No treatment group differed from SUC in the percentage of distance traveled (*p* > 0.05). SDTa5 and SDTa10 showed higher values than SMET12.5 (19.37%, *p* = 0.0278 and 20.82%, *p* = 0.0155, respectively; Figure 2C). For the percentage of time spent, SMET12.5 showed a lower value than SUC (−14.78%; *p* = 0.0017), whereas SMET25, SDTa5, and SDTa10 did not differ from SUC (*p* > 0.05). Both SDTa groups showed higher values than SMET12.5 (SDTa5: 13.21%, *p* = 0.0361 and SDTa10: 14.06%, *p* = 0.0223, respectively). No differences were detected between SDTa5 and SDTa10 (Figure 2D). Regarding ethological behaviors, grooming frequency differed among groups (Kruskal–Wallis: H(4) = 14.52; *p* = 0.0058), with lower grooming frequency in SDTa10 than in SMET12.5 (Dunn’s post hoc test, *p* = 0.0228; Figure 2E). No other differences in grooming frequency were detected, while grooming duration and rearing frequency and duration did not differ among groups (all *p* > 0.05).

In WAT-GR, no significant differences among groups were detected in total distance traveled or in the percentage of distance traveled, entries, or time spent in any OFT zone (all *p* > 0.05). Regarding ethological behaviors, grooming frequency differed among groups (Kruskal–Wallis: H(4) = 11.83; *p* = 0.0186); however, Dunn’s post hoc test identified no significant differences among groups. Grooming duration did not differ among groups (*p* > 0.05). Rearing frequency also showed an overall group effect (H(4) = 9.704; *p* = 0.0457), but no pairwise differences were detected after Dunn’s post hoc test. In contrast, rearing duration showed a significant group effect (H(4) = 11.51; *p* = 0.0214), with DTa10 showing a shorter rearing duration than CON (*p* = 0.0246), while no other significant differences were detected (Figure 2F).

#### 2.3.2. EPM

**Figure 3:**
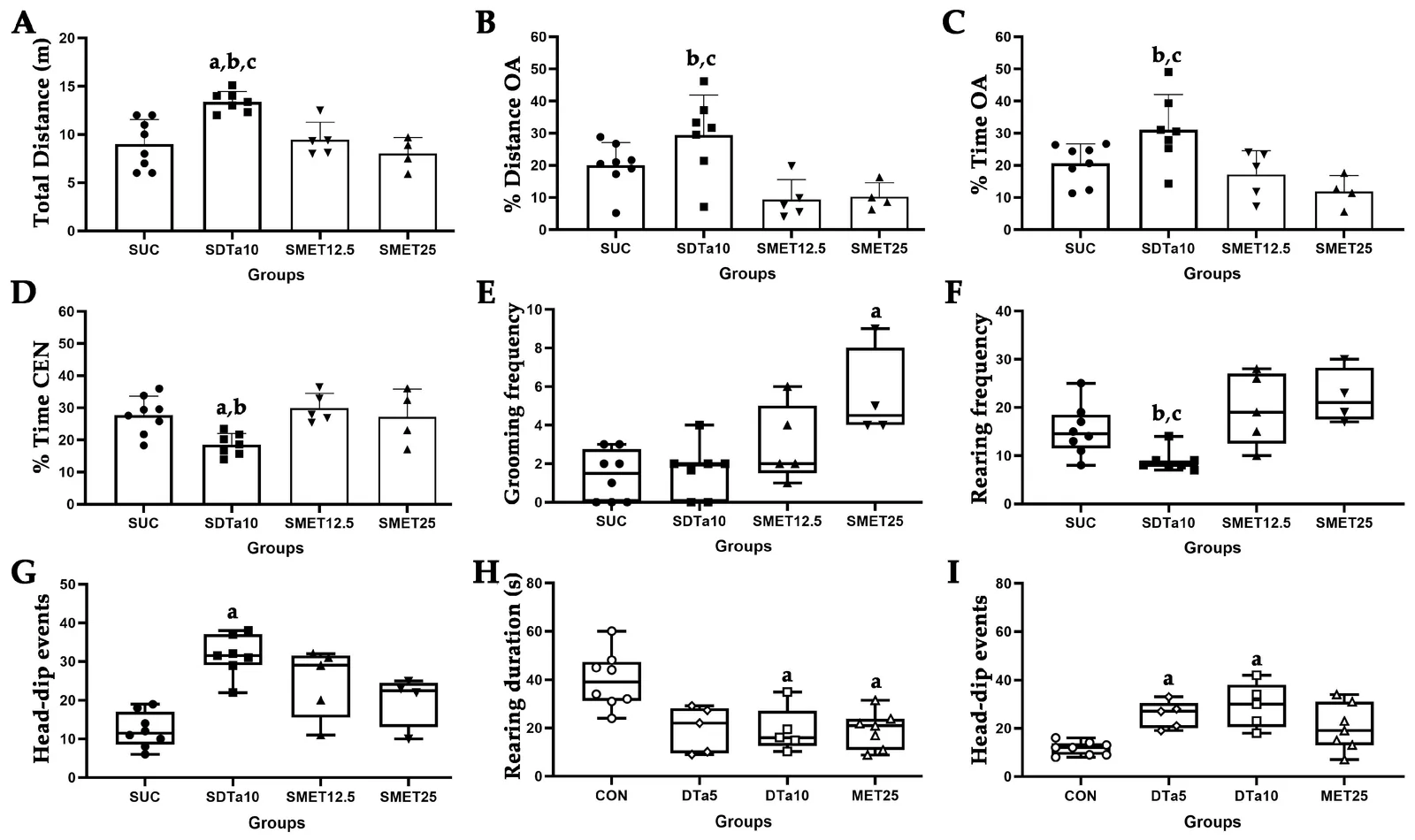
EPM behavioral parameters in SUC-GR: (**A**) Total Distance traveled, (**B**) % Distance in Open Arms (OA), (**C**) % Time in OA, (**D**) % Time in Center (CEN), (**E**) Grooming frequency, (**F**) Rearing frequency, and (**G**) Head dips events; and in WAT-GR: (**H**) Rearing duration and (**I**) Head dips events. Data in panel (**A**) were analyzed using Welch’s ANOVA followed by Dunnett’s T3 multiple comparisons test; panels (**B**–**D**) were analyzed using one-way ANOVA followed by Tukey’s post hoc test; and panels (**E**–**I**) were analyzed using the Kruskal–Wallis test followed by Dunn’s multiple comparisons test. Data are presented as mean ± SD for (**A**–**D**) and as median (interquartile range) for (**E**–**I**). Sample sizes were: SUC (*n* = 8), SDTa10 (*n* = 7), SMET12.5 (*n* = 5), SMET25 (*n* = 4), CON (*n* = 8), DTa5 (*n* = 5), DTa10 (*n* = 5), and MET25 (*n* = 7). **^a^** *p* < 0.05 vs. the corresponding control group (SUC in SUC-GR or CON in WAT-GR); **^b^** *p* < 0.05 vs. SMET12.5; **^c^** *p* < 0.05 vs. SMET25.

In SUC-GR, SDTa5 was excluded from the EPM analysis because only two animals successfully completed the test. Total distance traveled differed significantly among groups (Welch’s ANOVA: W(3, 8.829) = 15.43; *p* = 0.0007). Dunnett’s T3 multiple comparisons test showed that SDTa10 traveled a greater distance than SUC (48.89%; *p* = 0.0071), SMET12.5 (41.65%; *p* = 0.0241), and SMET25 (67.50%; *p* = 0.0186; Figure 3A). No difference was detected between the two metformin doses (*p* = 0.7530). In the open arms (OA), no significant differences among groups were observed in the percentage of entries (F(3,20) = 0.4842; *p* = 0.6970), whereas significant treatment effects were detected for the percentage of distance traveled (F(3,20) = 6.953; *p* = 0.0022) and the percentage of time spent (F(3,20) = 5.873; *p* = 0.0048). SDTa10 showed a greater percentage of distance traveled than SMET12.5 (216.25%; *p* = 0.0037) and SMET25 (187.24%; *p* = 0.0098). Similarly, SDTa10 showed a greater percentage of time spent in the OA than SMET12.5 (80.97%; *p* = 0.0342) and SMET25 (163.03%; *p* = 0.0050). No other significant differences were detected among groups for either parameter (Figure 3B,C). In the closed arms (CA), no significant differences among groups were observed in the number of entries (F(3,20) = 0.8032; *p* = 0.5067), distance traveled (Welch’s ANOVA: W(3,10.40) = 3.042; *p* = 0.0772), or time spent (F(3,20) = 1.240; *p* = 0.3215). In the central area (CEN), no significant differences among groups were observed in the number of entries (F(3,20) = 1.705; *p* = 0.1981), or distance traveled (F(3,20) = 2.099; *p* = 0.1325), whereas a significant treatment effect was detected for time spent (F(3,20) = 5.164; *p* = 0.0083). SDTa10 spent lower percentage of time than SUC (−33.06%; *p* = 0.0223) and SMET12.5 (−37.76%; *p* = 0.0123). No significant differences were detected between SUC and either metformin dose or between the two metformin doses (*p* > 0.05; Figure 3D). Regarding ethological behaviors, grooming frequency differed significantly among groups (Kruskal–Wallis: H(3) = 9.756; *p* = 0.0208). Dunn’s post hoc test showed a higher grooming frequency in SMET25 than in SUC (*p* = 0.0246), whereas no other significant differences were detected (Figure 3E). Grooming duration did not differ among groups (H(3) = 5.931; *p* = 0.1150). Rearing frequency also differed among groups (H(3) = 13.41; *p* = 0.0038), with SDTa10 showing a lower frequency than SMET12.5 (*p* = 0.0262) and SMET25 (*p* = 0.0076); no additional significant differences were detected (Figure 3F). Rearing duration did not differ among groups (H(3) = 4.929; *p* = 0.1771). Head-dip events differed significantly among groups (H(3) = 14.61; *p* = 0.0022), with SDTa10 showing a higher frequency than SUC (*p* = 0.0011); no other significant differences were detected (Figure 3G).

In WAT-GR, the MET12.5 group was excluded from the analysis, as only three animals completed the task. No significant differences among groups were detected in the percentage of distance traveled, time spent, or entries in the open arms, closed arms, or central area of the EPM (all *p* > 0.05). Regarding ethological behaviors, grooming frequency and duration did not differ among groups (*p* > 0.05). Rearing frequency showed a significant overall group effect (Kruskal–Wallis: H(3) = 9.802; *p* = 0.0203), although Dunn’s post hoc test identified no significant differences among groups. Rearing duration also showed a significant group effect (H(3) = 12.22; *p* = 0.0067), with MET25 and DTa10 showing shorter rearing durations than CON (*p* = 0.0219 and *p* = 0.0489, respectively); no additional significant differences were detected (Figure 3H). Similarly, a significant group effect was observed for head-dip frequency (H(3) = 13.06; *p* = 0.0045), with DTa5 and DTa10 showing higher frequencies than CON (*p* = 0.0348 and *p* = 0.0079, respectively); no other pairwise differences were detected (Figure 3I).

#### 2.3.3. NOL

In SUC-GR (SUC, *n* = 8; SMET12.5, *n* = 5; SMET25, *n* = 7; SDTa5, *n* = 7; SDTa10, *n* = 5), no significant differences among groups were detected in Total Exploration Time during T1 (one-way ANOVA: F(4,27) = 1.212; *p* = 0.3287). Exploration time did not differ between the two objects in any group (Wilcoxon matched-pairs tests: SUC, *p* = 0.5469; SMET12.5, *p* > 0.9999; SMET25, *p* = 0.2969; SDTa5, *p* = 0.1562; SDTa10, *p* = 0.1875), indicating no baseline preference for either object. Similarly, no group differences were observed during T2 in either the Discrimination Index (Kruskal–Wallis test: H(4) = 4.542; *p* = 0.3377) or Total Exploration Time (one-way ANOVA: F(4,27) = 0.5852; *p* = 0.6760).

In WAT-GR (CON, *n* = 10; MET12.5, *n* = 5; MET25, *n* = 6; DTa5, *n* = 6; DTa10, *n* = 6), Total Exploration Time also did not differ among groups during T1 (one-way ANOVA: F(4,28) = 2.013; *p* = 0.1199). Likewise, exploration time did not differ between the two objects at T1 in any group (Wilcoxon matched-pairs tests: CON, *p* = 0.6250; MET12.5, *p* = 0.6250; MET25, *p* = 0.0938; DTa5, *p* = 0.6875; DTa10, *p* = 0.6875), indicating no baseline preference for either object. During T2, neither the Discrimination Index (Kruskal–Wallis test: H(4) = 4.304; *p* = 0.3665) nor Total Exploration Time differed among groups (one-way ANOVA: F(4,28) = 1.214; *p* = 0.3270).

### 2.4. Ultra-High-Performance Liquid Chromatography–Photodiode Array–Orbitrap Tandem Mass Spectrometry (UHPLC–PDA–OT-MS/MS) Characterization of DTa

**Figure 4:**
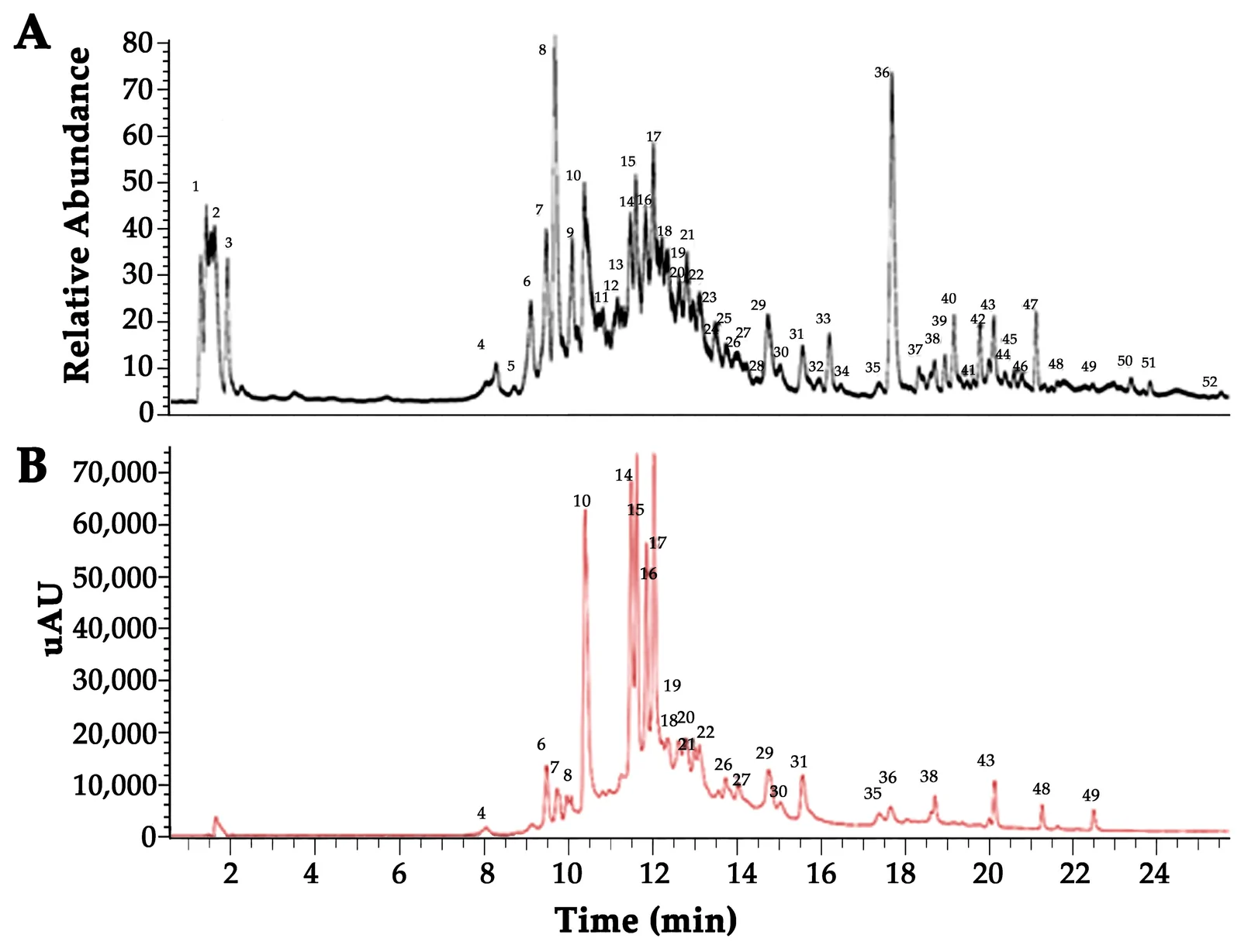
Representative UHPLC chromatograms of DTa. (**A**) Total ion chromatogram (TIC) acquired by UHPLC-PDA-OT-MS/MS in negative ionization mode and (**B**) UV chromatogram recorded at 280 nm. Numbered peaks correspond to the metabolites identified by UHPLC-PDA-OT-MS/MS and listed in Table S1.

**Figure 5:**
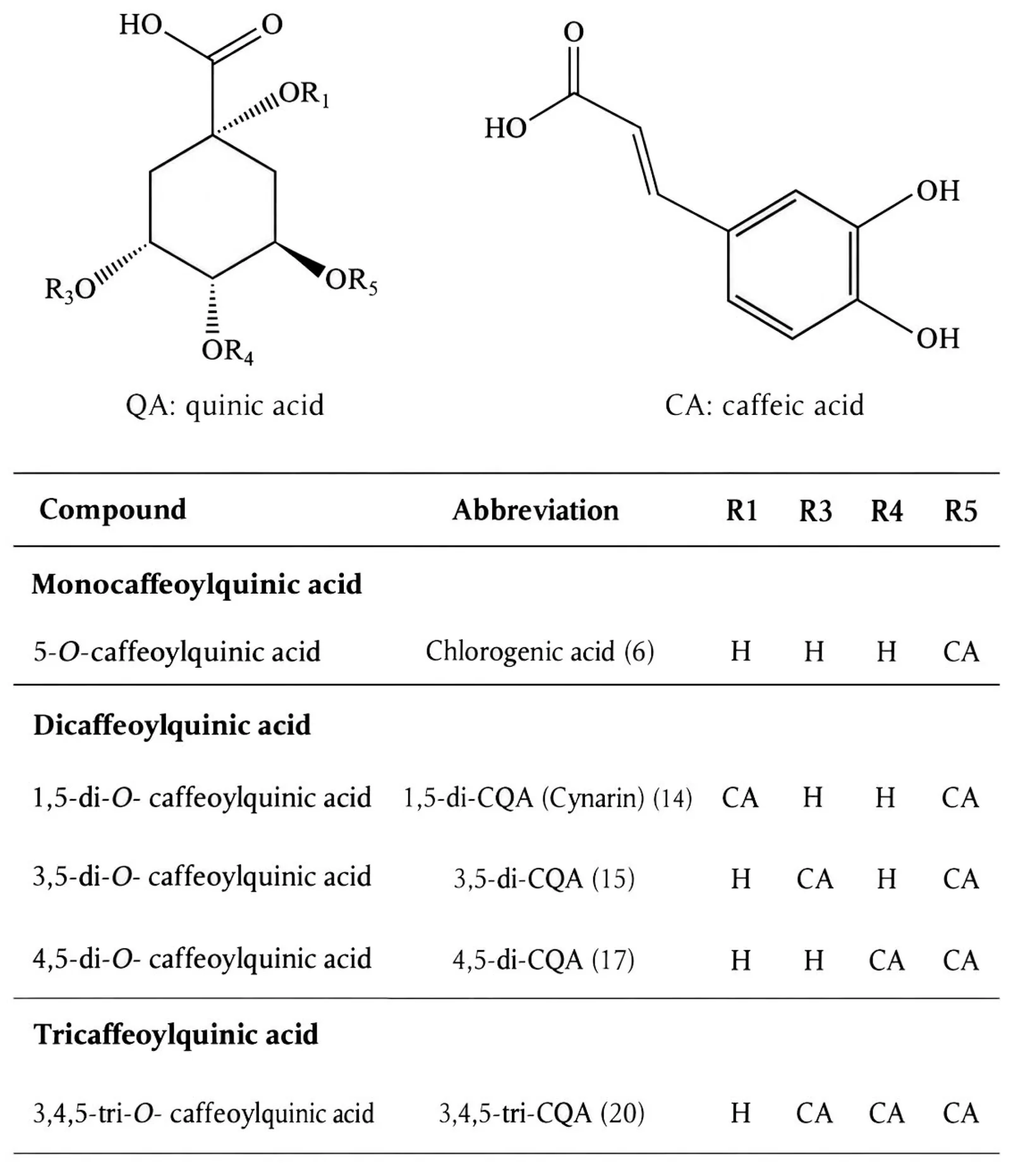
Molecular structures of some main representative mono, di and tri-caffeoylquinic acid identified in DTa using UHPLC-PDA-OT-MS/MS.

The UHPLC chromatograms (Figure 4) revealed the presence of fifty-one compounds in DTa, including phenolic acids, fatty acids, and characteristic eudesmane sesquiterpenoids, consistent with our previous phytochemical characterization [6]. The MS/MS properties of the detected metabolites, including retention times, molecular ions (negative ionization mode), and characteristic fragment ions, are summarized in Table S1. Representative major polyphenols identified in DTa are shown in Figure 5. Among the identified metabolites were eight phenolic acids, including chlorogenic acid (peak 6), vanillic acid (peak 8), three dicaffeoylquinic acid isomers (1,5-, 3,5-, and 4,5-diCQA; peaks 14, 15, and 17), 3,4,5-tricaffeoylquinic acid (peak 20), ginnalin A (peak 26), and a related tetracaffeoylquinic acid derivative (peak 30). Thirteen compounds were assigned as sesquiterpenes, most of them belonging to the characteristic eudesmane skeleton previously reported for *T. absinthioides*. These included 5,3,4,7-tetrahydroxypentosyl tessaric acid (peak 9), hymenoxynin (peak 11), ilicic acid (peak 16), eudesmane-4(15),11(13)-dien-12,5β-olide (peak 19), two dihydroxy-costic acid isomers (peaks 24 and 28), scorzonerin (peak 32), tessaric acid (peak 35) together with its isomers (peaks 38 and 40), 5-acetyl-3-hydroxy-4-reduced dihydro-costic acid (peak 39), 3-oxo-γ-costic acid (peak 42), γ-costic acid (peak 48), and α-costic acid (peak 50).

## 3. Discussion

The present study extends our previous findings by integrating the phytochemical characterization of *T. absinthioides* decoction with the evaluation of its metabolic and behavioral effects under both physiological and sucrose-fed conditions. The phytochemical profile of the DTa batch used in the present study, determined by UHPLC-PDA-OT-MS/MS, was highly consistent with our previous reports and with more recent analyses of decoctions prepared from the same geographical region, confirming chlorogenic acid, dicaffeoylquinic acid derivatives, vanillic acid, and characteristic eudesmane sesquiterpenoids as major constituents [5,6,18].

BW and beverage intake remained comparable among groups under both experimental conditions. The unchanged beverage intake in DTa and metformin-treated animals suggests that neither treatment affected consumption, consistent with our previous findings [6,16]. Together, these findings indicate that the metabolic and behavioral effects observed in the present study are unlikely to be explained by differences in treatment consumption.

Under sucrose-fed conditions, DTa effectively attenuated the alterations in glucose homeostasis associated with chronic sucrose consumption [19]. Both DTa and metformin improved glucose tolerance, as evidenced by their GTT profiles and reduced iAUC values; however, fasting glycemia was lower in DTa-treated animals than in SUC, an effect not observed with metformin, as previously shown [16]. Under normal conditions, fasting glycemia was lower in DTa10 than in CON, whereas DTa5 showed a similar trend that did not reach statistical significance. However, neither DTa concentration affected overall glucose tolerance, as indicated by the absence of significant treatment-related effects in the GTT and by iAUC values that did not differ from CON. Similarly, metformin did not affect either fasting glycemia or overall glucose tolerance under normal conditions. Thus, the metabolic effects of DTa were more pronounced under sucrose-fed conditions, suggesting that its actions on glucose homeostasis become more evident under metabolic challenge. The distinct pattern observed for fasting glycemia, with lower values in DTa-treated animals than in SUC but not in metformin-treated animals, suggests that the metabolic actions of DTa may involve additional mechanisms beyond those primarily associated with metformin. These effects may be related to the phytochemical composition of DTa, particularly the presence of chlorogenic acid and several caffeoylquinic acid derivatives, including 1,5-, 3,5-, and 4,5-diCQA. These phenolic compounds have demonstrated antihyperglycemic and insulin-sensitizing effects in different experimental models [20,21,22] and have been associated with improved glucose homeostasis through multiple complementary mechanisms, including enhanced insulin sensitivity, antioxidant activity, and modulation of glucose metabolism [23,24]. Likewise, several dietary polyphenols have been reported to improve glycemic control through complementary mechanisms [25,26,27]. Supporting this interpretation, similar metabolic effects have been reported for other members of the Asteraceae family. For instance, *Gynura divaricata* reduced fasting glucose and improved pancreatic function in a rodent model of type 2 diabetes [20]. Similarly, an infused drink prepared from *Taraxacum officinale* leaves and *Arctium lappa* roots attenuated hyperglycemia and insulin resistance in a fructose-induced metabolic syndrome model [28].

Behavioral analyses revealed distinct behavioral effects of DTa under sucrose-fed conditions, with additional changes in specific ethological behaviors under normal conditions. In the OFT, neither DTa concentration altered total distance traveled or the relative exploration of the different zones compared with SUC. In contrast, metformin exhibited a distinct exploratory profile, with both doses increasing the time spent in the intermediate zone and SMET12.5 reducing the time spent in the peripheral zone compared with SUC. Previously, using the two-bottle choice paradigm, we also found differences in anxiety-like responses following metformin treatment [16]. Grooming frequency was also lower in SDTa10 than in SMET12.5, although neither DTa concentration differed from SUC. However, because grooming may reflect both stress-related and self-regulatory behaviors depending on the experimental context, this finding should be interpreted with caution. In the EPM, SDTa10 exhibited greater total distance traveled and head-dip behavior compared with SUC, suggesting enhanced exploratory behavior and altered risk-assessment strategies. Moreover, SDTa10 showed greater exploration of the open arms, reflected by increased distance traveled and time spent in these arms compared with both metformin groups. SDTa10 also spent less time in the central area than SUC and SMET12.5 and showed lower rearing frequency than both metformin groups. Together, these findings indicate a distinct pattern of behavioral modulation by DTa10 in the EPM, involving locomotor, exploratory, and risk-assessment-related parameters. Under normal conditions, DTa did not alter classical anxiety-related parameters in either the OFT or EPM, but modulated specific ethological behaviors. DTa10 showed reduced rearing duration compared with CON in both tests. In addition, both DTa5 and DTa10 increased head-dip behavior compared to CON in the EPM. Notably, the latter effect was observed under both experimental conditions and, under normal conditions, occurred without changes in overall locomotor activity, supporting a consistent effect of DTa on exploratory and/or risk-assessment behavior. Interestingly, the behavioral effects observed under sucrose-fed conditions occurred alongside the improvement in glucose homeostasis. Although the present study was not designed to establish a causal relationship between metabolic and behavioral outcomes, this parallel response raises the possibility that the metabolic context may influence the neurobehavioral actions of DTa. This possibility is consistent with evidence linking high-sugar intake to alterations in brain function and emotional behavior [12,13]. Beyond the metabolic context, the behavioral effects of DTa may be related, at least in part, to its phytochemical profile. Chlorogenic acid has been reported to reduce anxiety-like behavior in rodents [29,30], whereas dicaffeoylquinic acid derivatives and other phenolic compounds have been associated with antioxidant and neuromodulatory activities [31,32]. Consistent with these observations, anxiolytic-like effects have also been reported for other members of the Asteraceae family. Experimental studies with *Tanacetum parthenium*, *Achillea millefolium*, and *Matricaria recutita* have demonstrated anxiolytic-like effects in rodent models, particularly in the EPM, further supporting the neurobehavioral potential of Asteraceae-derived phytochemicals [33,34,35]. Although the contribution of individual phytochemicals cannot be established from the present study, these compounds may collectively contribute to the modulation of exploratory and ethological behaviors observed with DTa. Regarding spatial recognition memory, no significant differences among groups were observed in the NOL test under either sucrose-fed or normal conditions. Total exploration time and discrimination index were comparable among groups, with no baseline preference for either object during familiarization. Thus, the behavioral effects observed with DTa were not accompanied by detectable differences in spatial recognition memory.

No consistent concentration-dependent pattern was observed between the 5% and 10% DTa preparations. Nevertheless, some behavioral effects were particularly evident with DTa10, especially under sucrose-fed conditions. Overall, the present findings broaden the evidence for the biological activity of *T. absinthioides* decoction and highlight the influence of the metabolic context on its effects. Together with its phytochemical profile, the metabolic and behavioral effects observed in the present study provide a basis for further studies aimed at identifying the bioactive constituents and mechanisms underlying its biological actions.

## 4. Materials and Methods

### 4.1. Plant Material

Aerial parts of *T. absinthioides* were collected in San Juan province, Argentina (2022, 31°37′44” S; 68°28′49” W); dried at room temperature according to traditional medicinal practices and stored protected from light and heat. A voucher specimen was deposited previously under voucher number IBT-TA-2 (Instituto de Biotecnología, Universidad Nacional de San Juan, Argentina). A 10% *w*/*v* decoction (DTa10) was prepared with dried and milled leaves and water purified with PSA equipment. After boiling for 30 min, the decoction was filtered and stored at −20 °C until use. The 5% *w*/*v* decoction (DTa5) was obtained by diluting DTa10 1:1 with purified water. A representative aliquot of DTa10 (100 mL) was lyophilized using LA-B3 freeze-drying unit (RIFICOR, Ciudad Autónoma de Buenos Aires, Argentina; yield: 1.13% *w*/*w*) and stored at −40 °C until UHPLC–PDA–OT-MS/MS analysis.

### 4.2. Metformin Preparation

A metformin hydrochloride tablet (500 mg; Química Montpellier S.A., Ciudad Autónoma de Buenos Aires, Argentina) was dissolved in 10 mL of drinking water to obtain a stock solution of 50 mg/mL. From this solution, doses of 12.5 and 25 mg/kg were administered according to BW. The solution was freshly prepared every 3 days.

### 4.3. Experimental Procedure

Male Sprague-Dawley rats (PND 21; *n* = 77) were housed under standard laboratory conditions in a temperature- and humidity-controlled vivarium, with a 12 h light/dark cycle and ad libitum access to food and water. All procedures concerning animal care and use were carried out according to the European Community Council Directive (86/609/EEC) and the guidelines of the National Institutes of Health Guide for the Care and Use of Laboratory Animals and approved by the ethical committee of IBYME (resolution No. 021/2021, CABA, Argentina). The animals were fed a normal diet (3.3 kcal/g, protein 18.2%, carbohydrates 56.9%, lipids 3.9%, vitamins and minerals 3%, fiber 5%, humidity 13%; Gepsa feeds, GrupoPilar SA, Argentina). At PND21, animals were allocated to one of two experimental settings according to sucrose exposure. One set of animals received sucrose throughout the study to evaluate the effects of DTa or metformin under sucrose-induced metabolic challenge (SUC-GR), whereas the other was maintained without sucrose to assess their effects under normal conditions (WAT-GR). In the SUC-GR, animals received sucrose 10% *w*/*v* (SUC, *n* = 10), sucrose + DTa 5% *w*/*v* (SDTa5, *n* = 8), sucrose + DTa 10% *w*/*v* (SDTa10, *n* = 8), sucrose + metformin 12.5 mg/kg (SMET12.5, *n* = 7), or sucrose + metformin 25 mg/kg (SMET25, *n* = 7). In the WAT-GR, animals received water (CON, *n* = 10), DTa 5% *w*/*v* (DTa5, *n* = 7), DTa 10% *w*/*v* (DTa10, *n* = 7), metformin 12.5 mg/kg (MET12.5, *n* = 6), or metformin 25 mg/kg (MET25, *n* = 7). All treatments and drinking regimens were maintained for 40 days. After this treatment period, all animals received water only, and metformin administration was discontinued. Metformin was administered orally on a daily basis throughout the treatment period. To adjust dosing, animals were weighed every three days. BW was recorded at baseline (PND21) and weekly until the end of the treatment period (PND61). Beverage consumption was recorded every other day between 09:00 and 10:00 a.m. throughout the 40-day treatment period. Animals were housed in cages containing 2–3 rats, and fluid intake was measured per cage; therefore, the cage was considered the experimental unit for intake analyses. At PND 61, all beverages were replaced with water. After 24 h, animals underwent a GTT. Behavioral assessments were conducted between PND 63-69 [16,19].

#### 4.3.1. GLU and GTT

GLU levels were determined from a drop of blood obtained from the tail vein of the 6 h-fasted animals, using test strips and a One Touch Ultra glucometer (LifeScan, Inc., a Johnson & Johnson^®^ company, Milpitas, CA, USA). The GTT and AUC were performed as previously described [16,19]. Briefly, glucose was administered i.p. at 2 g/kg BW, and blood glucose levels were measured at 0, 30, 60 and 120 min. Animals that did not exhibit the expected glycemic peak following glucose administration were excluded from the GTT analysis, as this likely reflected incomplete delivery of the glucose load during injection. Because fasting glucose levels differed among some treatment groups, iAUC was calculated relative to each animal’s baseline glucose value at 0 min to distinguish the glucose excursion following the glucose challenge from differences in fasting glycemia. The iAUC was calculated from glucose values obtained at 0, 30, 60, and 120 min using the trapezoidal method.

#### 4.3.2. Behavioral Tests

All behavioral tests were performed as previously described [16,19]. All apparatuses were illuminated by a 42 W light source positioned 100 cm above them and directed toward the ceiling, ensuring uniform light intensity across all areas. Animals were placed in the testing room and allowed to acclimate for at least 15 min prior to testing. All apparatuses were thoroughly cleaned with a 70% ethanol solution between trials to eliminate olfactory cues.

##### OFT and EPM

The OFT and EPM tests were performed and recorded as previously described [16,19]. The OF arena was digitally divided into three zones: central (Zone 1), intermediate (Zone 2) and peripheral (Zone 3). The EPM was divided into a Central area, Open arms, and Closed arms. The distance traveled, number of entries, and time spent in each zone were analyzed with the Any-maze tracking software version 7.6 (Stoelting Co., Wood Dale, IL, USA). Behavioral parameters were expressed as normalized values to account for differences in overall locomotor activity. Specifically, entries, distance traveled, and time spent in each zone were expressed as percentages of their corresponding total values (e.g., entries into a given zone as a percentage of total entries). Ethological behaviors, including Grooming [36] and Rearing [37] were manually scored by two independent observers blinded to the treatment conditions. In the EPM, Head-dip events (defined as the protrusion of the head over the edge of an open arm) were also manually quantified as a measure of risk assessment [38]. In the EPM, animals that fell from the apparatus before completing the test were excluded from the analysis. Groups with insufficient sample size (*n* < 4) were not included in statistical comparisons.

##### NOL

The NOL test was performed as previously described [16,19]. Briefly, the test consists of two trials. In the first trial (Trial 1), the animal is placed in an open-field arena (a dark open box made of wood) containing two identical objects positioned equidistantly, and allowed to explore for 5 min. Animals that did not reach a total exploration time of 20 s were excluded from the analysis and did not proceed to the subsequent trial. After a 2 h intertrial interval, the second trial (Trial 2) was conducted, in which one object was relocated to a novel position (N), while the other remained in its original, familiar location (F). Animals were again allowed to explore for 5 min. Total Exploration Time was quantified in both trials. In Trial 1, the time spent exploring each object was compared within each group to assess the absence of inherent object preference. In Trial 2, Total Exploration Time and the Discrimination Index were calculated. The Discrimination Index was defined as (tN − tF)/(tN + tF), where tN and tF represent the time spent exploring the N and F objects, respectively. Positive DI values indicate preference for the novel object, whereas values close to zero or negative indicate no preference or preference for the familiar object, respectively.

### 4.4. UHPLC-PDA-OT-MS/MS

The phytochemical profile of DTa was analyzed using an ultra-high-performance liquid chromatography system (Thermo Dionex Ultimate 3000, Thermo Fisher Scientific, Waltham, MA, USA) equipped with a photodiode array (PDA) detector and controlled by Chromeleon 7.2 software (Thermo Fisher Scientific, Waltham, MA, USA), coupled to a Q-Exactive Focus Orbitrap mass spectrometer (Thermo Fisher Scientific, Bremen, Germany). Lyophilized DTa10 (5 mg) was dissolved in 2 mL of methanol, filtered through a 200 µm PTFE (polytetrafluoroethylene) membrane filter, and a 10 µL aliquot was injected into the UHPLC system. Sample preparation and analytical procedures were performed as previously described [6].

### 4.5. Liquid Chromatography and Mass Spectography Parameters

Chromatographic separation was carried out on an Acclaim C18 UHPLC column (150 mm length × 4.6 mm diameter, 2.5 µm particle size; Thermo Fisher Scientific, Bremen, Germany) maintained at 25 °C. Detection wavelengths were set at 254, 280, 330, and 354 nm, while photodiode array detectors were set from 200 and 800 nm. The mobile phase consisted of 1% aqueous formic acid (solvent A) and acetonitrile containing 1% formic acid (solvent B). The gradient program started at 5% B (0–5 min), increased to 30% B over 10 min, was maintained at 30% B for 15 min, increased to 70% B over 5 min, held at 70% B for 10 min, and finally returned to the initial conditions for column equilibration before the next injection. The flow rate was 1.0 mL min^−1^, and the injection volume was 10 µL. Standards, dissolved in methanol, and DTa samples were maintained at 10 °C in the autosampler throughout the analysis. The UHPLC system was coupled to the mass spectrometer through a heated electrospray ionization source (HESI II). Instrumental parameters were optimized as previously reported [6]. Briefly, sheath gas flow was set to 75 arbitrary units, auxiliary gas flow to 20 units, capillary temperature to 400 °C, auxiliary gas heater temperature to 500 °C, spray voltage to 2500 V (ESI−), and S-lens RF level to 30. Full-scan spectra were acquired in both positive and negative ionization modes over an *m*/*z* range of 100–1000 at a resolving power of 70,000 FWHM (*m*/*z* 200). Automatic gain control (AGC) was set to 3 × 10^6^, and the maximum injection time was 200 ms.

Nitrogen (purity > 99.999%), supplied by a Genius NM32LA generator (Peak Scientific, Billerica, MA, USA), was used as sheath, auxiliary, collision, and damping gas. The Orbitrap mass analyzer was calibrated daily in both positive and negative ionization modes to ensure a mass accuracy of 5 ppm. For positive mode, calibration was performed using a mixture of caffeine (1 mg/mL, 20 µL) and N-butylamine (1 mg/mL, 100 µL). For negative mode, sodium dodecyl sulfate (1 mg/mL, 100 µL) and sodium taurocholate (1 mg/mL, 100 µL; Sigma-Aldrich, Darmstadt, Germany) were used. Ultramark 1621 (Alfa Aesar, Stevensville, MI, USA) served as the reference compound. Calibration solutions were prepared in a mixture of acetic acid (100 µL), acetonitrile (5 mL), and water:methanol (1:1, 5 mL; Merck, Santiago, Chile), and infused (20 µL) using a Chemyx Fusion syringe pump (Thermo Fisher Scientific, Bremen, Germany). Instrument control and data processing were performed using Q Exactive 2.0 SP2, Xcalibur 2.3, and Trace Finder 3.2 software (Thermo Fisher Scientific, Bremen, Germany), as previously described [6].

### 4.6. Statistical Analysis

Statistical analyses were performed using GraphPad Prism (GraphPad Software Inc., version 10, San Diego, CA, USA). Data distribution was assessed using the Shapiro–Wilk test. Homogeneity of variances was assessed using the Brown-Forsythe test. For normally distributed data, ordinary one-way ANOVA followed by Tukey’s multiple comparisons test was used when homogeneity of variances was met; when this assumption was violated, Welch’s ANOVA followed by Dunnett’s T3 multiple comparisons test was applied. BW, beverage intake over time, and glucose levels during the GTT were analyzed using two-way RM ANOVA followed by Šídák’s multiple comparisons test when appropriate. When the assumption of sphericity was violated, the Greenhouse-Geisser correction was applied. Behavioral parameters analyzed using non-parametric statistics, including Grooming, Rearing, and Head-dip behaviors, were analyzed using the Kruskal–Wallis test followed by Dunn’s multiple comparisons test when appropriate. In the NOL familiarization trial (T1), exploration times for the two objects were compared within each group using the Wilcoxon matched-pairs test. The Discrimination Index was analyzed using the Kruskal–Wallis test. In all cases, data are presented as mean ± standard deviation (SD) for parametric variables or median (interquartile range) for non-parametric variables. Differences were considered statistically significant at *p* < 0.05.

## 5. Conclusions

The present study integrates the phytochemical characterization of *T. absinthioides* decoction with the evaluation of its metabolic and behavioral effects. Under sucrose-fed conditions, DTa was associated with lower fasting glycemia and improved glucose tolerance, as supported by reduced iAUC values. Behavioral analyses revealed that, under sucrose-fed conditions, DTa modulated exploratory and risk-assessment behavior, with effects particularly evident in the EPM. Under normal conditions, DTa also modulated specific ethological behaviors, including increased head-dipping, while classical anxiety-related parameters remained unaffected. No differences in spatial recognition memory were detected among groups within either the sucrose-fed or normal condition. Overall, the integration of the phytochemical characterization with the biological findings supports the potential of *T. absinthioides* as a source of bioactive phytocompounds capable of modulating metabolic and behavioral responses, particularly under conditions of metabolic challenge. Future studies should focus on identifying the bioactive compounds responsible for these effects and elucidating their underlying mechanisms of action.

## Data Availability

The original contributions presented in this study are included in the article. Further inquiries can be directed to the corresponding author.

## Supplementary Materials

The following supporting information can be downloaded at: https://www.mdpi.com/article/10.3390/plants15182872/s1. Figure S1: Body weight (BW) and beverage intake in SUC-GR (A,B: SUC, SDTa5, SDTa10, SMET12.5 and SMET25) and WAT-GR (C,D: CON, DTa5, DTa10, MET12.5 and MET25). Table S1: High resolution UHPLC PDA-Q orbitrap identification of metabolites from DTa.

## Abbreviations

The following abbreviations are used in this manuscript:

BW	Body Weight
CA	Closed Arms
CEN	Center
CON	Control Group
diCQA	dicaffeoylquinic acid
DTa	Decoction of *T. absinthioides*
DTa10	Decoction 10% *w*/*v* of *T. absinthioides* Group
DTa5	Decoction 5% *w*/*v* of *T. absinthioides* Group
EPM	Elevated Plus Maze
GLU	Basal Glucose
GTT	Glucose Tolerance Test
HDL-c	High-Density Lipoprotein Cholesterol
iAUC	Incremental Area Under Curve
MET12.5	Metformin 12.5 mg/kg Group
MET25	Metformin 25 mg/kg Group
NOL	Novel Object Location
OA	Open Arms
OFT	Open Field Test
RM-ANOVA	Repeated Measure ANOVA
SD	Standard Deviation
SDTa10	Sucrose 10% *w*/*v* + Decoction 10% *w*/*v* of *T. absinthioides* Group
SDTa5	Sucrose 10% *w*/*v* +Decoction 5% *w*/*v* of *T. absinthioides* Group
SMET12.5	Sucrose 10% *w*/*v* + Metformin 12.5 mg/kg Group
SMET25	Sucrose 10% *w*/*v* + Metformin 25 mg/kg Group
SUC	Sucrose 10% *w*/*v* Group
SUC-GR	sucrose-induced metabolic challenge condition
T1	Trial 1
T2	Trial 2
UHPLC-PDA-OT-MS/MS	Ultra-High Performance Liquid Chromatography-Photodiode Array Detector Orbitrap Mass Spectrometry
WAT-GR	normal conditions
