# Supplementary material for: Tessaria absinthioides (Hook. & Arn.) DC. (Asteraceae) Decoction vs. Metformin: Effects on Glucose Homeostasis and Behavior Under Chronic Sucrose Exposure
Source: Plants (Basel). 2026 Sep 19;15(18):2872. doi: 10.3390/plants15182872 (PMC13611746; doi:10.3390/plants15182872)
Supplement: Supplementary file 1 [file plants-15-02872-s001.zip › plants-4498322-supplementary.pdf]

Supplementary Materials

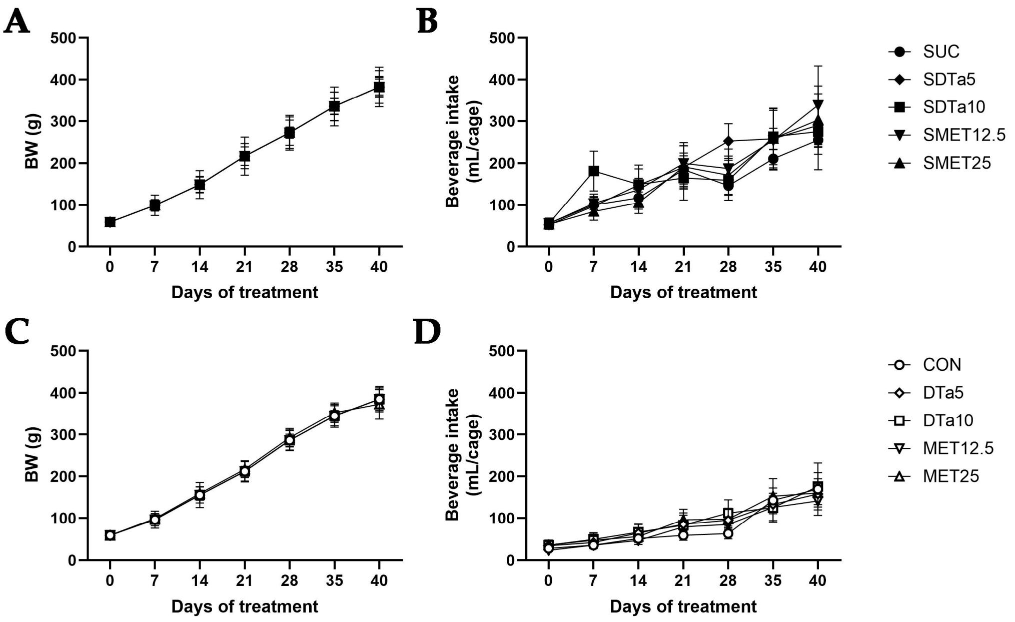

Figure S1. Body weight (BW) and beverage intake in SUC-GR (**A**,**B**: SUC, SDTa5, SDTa10, SMET12.5 and SMET25) and WAT-GR (**C**,**D**: CON, DTa5, DTa10, MET12.5 and MET25). Data were analyzed using two-way RM-ANOVA followed by Šídák's multiple comparisons test. For BW, sample sizes were: SUC (n = 10), SDTa5 (n = 8), SDTa10 (n = 8), SMET12.5 (n = 7), SMET25 (n = 7), CON (n = 10), DTa5 (n = 7), DTa10 (n = 7), MET12.5 (n = 6), and MET25 (n = 7). For beverage intake, the cage was considered the experimental unit (2 - 3 animals per cage). Sample sizes were: SUC (n = 5), SDTa5 (n = 3), SDTa10 (n = 3), SMET12.5 (n = 3), SMET25 (n = 3), CON (n = 5), DTa5 (n = 3), DTa10 (n = 3), MET12.5 (n = 3), and MET25 (n = 3). Data are presented as mean ± SD.

Table S1. High resolution UHPLC PDA-Q orbitrap identification of metabolites from DTa.

Peak #	Retention time (min.)	UV max	Tentative identification	Elemental com-position [M-H]	Measured mass (m/z)	Theoretical mass (m/z)	Ac curacy (ppm)	MS^n ions (ppm)
**1**	1.21	-	Quinic acid *	$C_7H_{11}O_6^-$	191.05579	191.05501	4.03	144.00844
**2**	1.31	-	Manoheptulose*	$C_7H_{13}O_7^-$	209.06633	209.06558	3.37	153.01857
**3**	1.82	-	Citric acid*	$C_6H_7O_7^-$	191.01863	191.01939	3.76	144.00844
**4**	7.26	-	Unknown	$C_{16}H_{11}O_{15}N_3^-$	365.01859	365.01847	0.23	
**5**	8.47	330	Unknown	$C_4H_7O_{12}^-$	246.99167	246.99320	-6.2	152.01080
**6**	8.73	239-320	5-O-caffeoylquinic acid (chlorogenic acid)*	$C_{16}H_{17}O_9^-$	353.08671	353.08786	3.86	275.0235, 191.05481 (quinic acid), 707.18115 (2M-H)
**7**	9.55	223	3-Hydroxysuberic acid	$C_8H_{13}O_5^-$	189.07645	189.07575	3.69	

**8**	10.03	330	Vanillic acid*	$C_9H_7O_4^-$	179.03465	179.03389	4.28	135.04436
**9**	10.07	283	5,3,4,7 tetrahydroxypen-tosyl tessaric acid	$C_{20}H_{31}O_{10}^-$	431.19241	431.19117	2.87	311.11367, 135.04433
**10**	10.36	330	Unknown	$C_{10}H_9O_{15}N_3^-$	411.00293	411.00282	0.32	
**11**	10.43	288-346	Hymenoxynin	$C_{21}H_{34}O_9^-$	429.21313	429.21191	2.85	267.21184 (M-hexosemoiety)
**12**	11.12	330	Unknown	$C_{15}H_7O_{11}N^-$	377.00092	377.00136	-1.18	
**13**	11.48	330	Unknown	$C_{10}H_9O_{15}N_3^-$	411.00296	411.00282	0.34	
**14**	11.62	239-320	1, 5-di-O-caffeoyl quinic acid (cynarin)	$C_{25}H_{23}O_{12}^-$	515.11945	515.11840	2.02	191.05551 (quinic acid), 179.03429
**15**	11.82	239-320	3, 5-di-O-caffeoyl quinic acid*	$C_{25}H_{23}O_{12}^-$	515.11951	515.11840	2.14	191.05562 (quinic acid), 179.03429
**16**	11.90	255-354	Ilicic acid*	$C_{15}H_{23}O_3^-$	251.16516	251.16417	4.5	233.15470 (M-H_2O), 207.17544 (M-CO_2),171.95076
**17**	12.00	239-320	4, 5-di-O-caffeoyl quinic acid	$C_{25}H_{23}O_{12}^-$	515.11840	515.11945	2.14	191.05551 (quinic acid), 179.03429
**18**	12.20	335	Bruceantin	$C_{28}H_{36}O_{11}^-$	547.21739	547.21832	1.70	
**19**	12.21	255-365	Eudesmane 4(15), 11(13)-dien-12, 5βolide*	$C_{15}H_{19}O_3^-$	247.13380	247.13287	3.77	205.15968 (M-CO_2),149.09645
**20**	12.57	289-329	3,4,5-tri-O-caffeoylquinic acid	$C_{34}H_{39}O_{15}^-$	677.15033	677.15119	14.30	515.11963 (Di-CQA), 191.05561(quinic acid)
**21**	12.81	278	Unknown	$C_{30}H_{33}O_{15}N_{10}^-$	773.13715	773.21214	-0.08	
**22**	12.96	278	Unknown	$C_{26}H_8ON_{10}^-$	476.08734	476.08771	-0.76	
**23**	13.21	278	Unknown	$C_{12}HO_{16}N_{14}^-$	953.17645	953.17632	-0.98	476.08722
**24**	13.46	278	3,4-Dihydroxy-costic acid*	$C_{15}H_{21}O_4^-$	265.14459	265.14344	4.32	247.13395 (M-H_2O)
**25**	14.04	278	Unknown	$C_{28}H_{23}O_{12}N_9^-$	677.15039	677.14741	4.40	
**26**	14.70	323	Ginnalin A	$C_{20}H_{19}O_{13}^-$	467.08200	467.08202	-0.03	249.08006
**27**	14.29	213, 287, 326	1-O-Caffeoyl-5-O-feruloyl-quinic acid	$C_{26}H_{25}O_{12}^-$	529.13405	529.13519	2.15	191.05577 (quinic acid), 134.03664
**28**	16.20	278	3,5-Dihydroxy-costic acid	$C_{15}H_{21}O_4$	265.14456	265.14344	4.24	247.13392(M-H_2O)
**29**	16.72	225	10-Undecenoic acid*	$C_{43}H_{35}O_{18}^-$	227.12779	227.11839	2.64	183.13847
**30**	15.55	265-329	Tetra caffeoylquinic acid	$C_{43}H_{35}O_{18}^-$	839.17999	839.18289	3.43	191.05552 (quinic acid), 179.03423

**31**	15.93	335	Sambucinol	$C_{15}H_{21}O_4^-$	265.14344	265.14453	4.13	
**32**	15.94	235	Scorzonerin	$C_{30}H_{36}O_{11}^-$	571.21872	571.21739	1.63	467.08185, 327.21765
**33**	16.45	335	2,3,4-Trihydroxyoctadeca-2,4-dienoic acid	$C_{18}H_{31}O_5^-$	327.21660	327.21783	3.77	183.13832
**34**	16.48	222	Trihydroxy-octadecadienoic acid	$C_{18}H_{31}O_5^-$	327.21790	327.21660	3.95	283.22787(M-CO_2)
**35**	17.69	283	Tessaric acid*	$C_{15}H_{19}O_3^-$	247.13379	247.13287	3.71	205.15979 (M-CO_2), 149.09644
**36**	18.34	222	Trihydroxy-octadecaenoic acid	$C_{18}H_{33}O_5^-$	329.23225	329.23364	4.22	285.24352(M-CO_2)
**37**	18.69	335	Jaceidin	$C_{18}H_{15}O_8^-$	359.07614	359.07745	3.64	
**38**	19.09	283	Tessaric acid isomer	$C_{15}H_{19}O_3^-$	247.13383	247.13287	3.90	205.15972 (M-CO_2),163.11223
**39**	19.25	218	5-Acetyl, 3-hydroxy-4 dihydrocostic acid	$C_{17}H_{25}O_5^-$	309.17090	309.16965	4.03	291.16019(M-H_2O), 267.16018(M-acetylmoiety)152.08374
**40**	19.47	283	Tessaric acid isomer	$C_{15}H_{19}O_3^-$	247.13383	247.13287	3.90	205.15979 (M-CO_2),162.01357
**41**	19.80	320	Artelasticin	$C_{30}H_{33}O_6^-$	489.24282	489.24405	2.5	245.99947
**42**	19.96	283	3-oxo-gamma costic acid*	$C_{15}H_{19}O_3^-$	247.13383	247.13287	3.90	231.13903, 233.11812, 219.13902, 215.00955, 149.09644
**43**	20.14	335	Chrysosplenetin	$C_{19}H_{17}O_8^-$	373.09313	373.09317	3.68	310.40393
**44**	20.39	335	3-Acetyl,5-hydroxy-4 dihydro costic acid	$C_{17}H_{25}O_5^-$	309.16965	309.17099	4.33	
**45**	21.12	335	Geranylpropionate	$C_{15}H_{21}O_2^-$	209.15361	209.15439	3.73	
**46**	21.53	218	Eupatorin	$C_{18}H_{15}O_8^-$	343.08258	343.08123	-4.11	329.06663 (M-CH_3),315.0533 (M-2CH_3), 313.03531
**47**	22.53	218	Unknown	$C_{13}H_{27}O_8^-$	311.16876	311.17004	-4.11	
**48**	23.41	335	Gamma costic acid*	$C_{15}H_{21}O_2^-$	233.15453	233.15451	3.87	215.00955, (M-H_2O)205.15973(M-CO)
**49**	23.41	335	Gamma costic acid isomer	$C_{15}H_{21}O_2^-$	233.15453	233.15361	3.94	215.00955, (M-H_2O)205.15973(M-CO)
**50**	23.97	335	Alpha Costic acid	$C_{15}H_{21}O_2^-$	233.15451	233.15361	3.87	215.00953,(M-H_2O)205.15965 (M-CO)
**51**	24.61	320	2-Hydroxydocosanoic acid	$C_{22}H_{43}O_3^-$	355.32067	355.32175	3.02	
**52**	24.78	-	Unknown	$C_{14}H_{29}O_8^-$	325.18569	325.18436	-4.11	311.16861

*Identified by spiking experiments with authentic standards.
